# Doctor Retention: A Cross-sectional Study of How Ireland Has Been Losing the Battle

**DOI:** 10.34172/ijhpm.2020.54

**Published:** 2020-04-22

**Authors:** Ruairi Brugha, Nicholas Clarke, Louise Hendrick, James Sweeney

**Affiliations:** ^1^RCSI Division of Population Health Sciences, Royal College of Surgeons in Ireland, Dublin, Ireland.; ^2^School of Psychology, Dublin City University, Dublin, Ireland.; ^3^National Doctors Training and Planning, Health Service Executive, Dublin 8, Ireland.; ^4^Department of Mathematics & Statistics, University of Limerick, Limerick, Ireland.

**Keywords:** Workforce, Doctor Retention, Migration, WHO Global Code, Ireland

## Abstract

**Background:** The failure of some high-income countries to retain their medical graduates is one driver of doctor immigration from low- and middle-income countries. Ireland, which attracts many international medical graduates, implemented a doctor retention strategy from early 2015. This study measures junior doctors’ migration intentions, the reasons they leave and likelihood of them returning. The aim is to identify the characteristics and patterns of doctors who plan to emigrate to inform targeted measures to retain these doctors.

**Methods:** A national sample of 1148 junior hospital doctors completed an online survey in early 2018, eliciting their experiences of training and working conditions. Respondents were asked to choose between the following career options: remain in Ireland, go and return, go and stay away, or quit medicine. Bivariate analyses and a two-stage multivariable analysis were used to model the factors associated with these outcomes.

**Results:** 45% of respondents planned to remain in Ireland, 35% leave but return later, 17% leave and not return; and 3% to quit medicine. An intention to go abroad versus remain in Ireland was independently associated (*P* <.05) with the doctor being under 30 years (odds ratio [OR]=1.09 per year under 30), a non-European Union (EU) national (OR=1.54), a trainee (OR=1.50), and with hospital specialization, especially in Anesthesiology (OR=5.09). Respondents were more likely to remain if they had experienced improvements in supervision and training costs. Intention to go abroad and not return versus go and return was independently associated (*P* <.05) with: age over 30 years (OR=1.16 per year over 30); being a non-EU (OR=9.85) or non-Irish EU (OR=3.42) national; having trained through a graduate entry pathway (OR=2.17), specializing in Psychiatry (OR=4.76) and reporting that mentoring had become worse (OR=5.85).

**Conclusion:** Ireland’s doctor retention strategy has not addressed the root causes of poor training and working experiences in Irish hospitals. It needs a more diversified retention strategy that addresses under-staffing, facilitates circular migration by younger trainees who choose to train abroad, identifies and addresses specialty-specific factors, and builds mentoring linkages between trainees and senior specialists.

## Background

The damaging impact of the outward migration of health personnel on the health systems of low-income countries was the principle rationale for the World Health Organization (WHO) Global Code on the International Recruitment of Health Personnel.^1^ In the case of doctors, one root cause and driver of migration is the failure of some high-income countries to produce sufficient numbers of medical graduates, and/or their failure to retain their domestic medical workforce. Studies measuring the intentions, levels and reasons for the outward migration of doctors from high-income countries continue to grow: Portugal reported 55% of recent medical graduates intending to migrate^2^; the United Kingdom an estimated 60%,^3^ and Germany 30%.^4^ In Romania, almost all (85% of) medical students surveyed between 2013 and 2015 planned to emigrate on graduation.^5^ The costs of employing doctors, unsatisfactory working conditions, and perceptions of better training and career opportunities abroad are driving doctor emigration.^6^ In Greece, low job satisfaction, fears of unemployment and a lack of standardized training are reported as reasons for emigration.^7,8^ Austria has seen 30% of its graduates emigrating due to negative perceptions of postgraduate training, low basic salaries, burdensome administrative tasks and long working hours.^9,10^ More generally, studies and reports from the United Kingdom, the United States and Ireland report poor work-life balance, ill health, burnout and the negative impact of training structures on early career doctors’ personal and family lives.^11-14^

There are 2 limitations in most of the research literature^2-13^: while studies report reasons for outward migration and propose or report retention measures that are in place, they usually do not differentiate the career intentions of the different types of doctors. Secondly, they do not evaluate the effectiveness of the retention measures. An exception is the General Medical Council’s 2018 report on the state of medical education and practice in the United Kingdom,which reported that 28% of doctors aged 18-34 years were considering practicing abroad.^11^ This paper reports the migration intentions of different categories of early career doctors working in Ireland, linking these to their training and working experiences.

Ireland is in the top 3 high-income countries for reliance on international recruitment, having recruited several thousand doctors from South-East Asia and Africa in the last 20 years.^15,16^ The countries where most currently registered graduates qualified, after Ireland (58%), are: Pakistan (8%), Sudan (4%), and South Africa and the United Kingdom, each 3.6%.^17^ A 2006 national report recommended an almost doubling of Ireland’s domestic production of doctors from 370 to 725, so as to achieve medical workforce self-sufficiency, through establishing new 4 year graduate entry medicine (GEM) programs (enrolling medical school entrants who have a graduate degree) to run alongside longstanding 5-6 year direct entry programs (medical school entrant normally direct from secondary school).^18^ Ireland produces the most doctors among the Organization for Economic Cooperation and Development (OECD) countries, with 22.1 medical graduates compared with an OECD average of 12.5 per 100 000 population; although at 2.9 per 1000 population (OECD average, 3.4), Ireland is below average for practicing doctors.^19^

Almost all non-European Union (EU)graduates leave Ireland after graduation because preference is given to EU nationals when filling internship posts, which is the compulsory year of hospital practice after graduation. In July 2017, 679 Irish and 26 EU nationals who had qualified in Ireland, and 28 EU nationals who had qualified overseas, were offered internship posts.^20,21^ Following completion of the intern year, doctors can compete to enter a 2-4 year Basic Specialist Training (BST) program, followed by competitive entry to a 4-6 year Higher Specialist Training (HST) program, successful completion of which allows doctors join the specialist training register and compete for permanent consultant and general practitioner posts.^20^ Some specialties have introduced ‘run-through’ programs, whereby trainees progress seamlessly from BST to HST. A 2014 survey of Irish-trained doctors who had emigrated,^22^ and a 2016 survey of non-consultant hospital doctor (NCHD) trainees,^23^ both reported high levels of dissatisfaction with working conditions, training opportunities and career prospects, as reasons for leaving Ireland. NCHDs in Ireland correspond to junior hospital doctors in the United Kingdom and other countries. They comprise interns and doctors in BST/HST/Run through training programs (trainees); and currently an equal number of NCHDs in non-training scheme posts, which now account for 50% of post-internship NCHD posts.^20^

Accurate estimates of emigration are unavailable, although analyses of 4 years of medical council registration data in Ireland showed average exit rates of 8.5% among 25-34 year olds and 6.6% among 35-44 year olds.^24^ An analysis of routine medical register and visa data in 5 popular Anglophone destination countries, covering 2008-2014, suggested that up to 3800 doctors had left Ireland, equivalent to the numbers of Irish/EU graduates during this period,^25^ eliminating the benefits to Ireland of increased domestic production. Earlier qualitative work highlighted the welcome towards Irish trained doctors and the ease with which they gain entry to specialist training programs abroad.^22^ Doctor emigration from Ireland continues unabated.^26^ The propensity and reasons for large scale emigration of doctors from Ireland were reported by Gish in the later 1960s, including^27^: “staffing structure in the hospitals … lack of advancement, the desire for further specialization, for better pay ….” While the emigration drivers are longstanding,^28^ the scale of emigration since 2008 is no longer compatible with the assumption that migrating doctors will return home after undertaking additional training abroad.^29^

Our 2018 survey of Irish interns reported high levels (57%) planning to go but return later; and only 7% of interns planned to go and not return.^30^ However, intention-to-return rates among emigrated doctors fall over time, if they see little evidence of improvement in training and working conditions at home; and as they put down professional and family roots in destination countries.^31,32^ Many Irish doctors, whether working in Ireland or abroad, now envisage their ultimate careers being abroad.^22,23^ Additional analysis of our survey of 588 trainees showed that by 2016, 19 (25%) of 57 interns and 42 (28%) of 152 doctors in HST who had been surveyed in 2014,^23^ had left Ireland.^33^ Irish doctors are reportedly recruited for permanent specialist posts abroad, as they are completing specialist training in destination countries and awaiting suitable posts in Ireland.^33^ This pointed to the need to understand the migration intentions of early career doctors by training status, stage and specialty.

A series of national reports in the last 15 years have recommended improving working conditions, training structures and career opportunities for early career doctors in Ireland.^34-36^ The 2014 Strategic Review of Medical Training and Career Structures,^37^ tasked with “improving graduate retention in the public health system,” stressed the need for recommendations to result in tangible improvements in NCHDs’ day-to-day working lives, noting the “imbalance between training needs and service requirements.” Implementation of its 25 recommendations, which largely focused on working conditions and training opportunities, has been monitored by a multi-stakeholder group over the 5 years since January 2015.^38^ A draft tenth progress report was circulated in September 2019^39^ in which, as in earlier reports, NCHDs reported that there had been little or no progress in 4 critical areas that are evaluated in this paper: protected training time, non-core task reallocation, postgraduate training costs; and the additional challenges faced by non-training scheme doctors.

Following consultations with key national medical workforce agencies, the authors of this paper designed a study to elicit the views of NCHDs regarding whether or not they had seen improvements in these critical dimensions of their working and training lives, and in other factors shown to be determinants of doctors’ intentions or reasons for emigrating.^22,23^ This paper reports the results of this cross-sectional survey of NCHDs, conducted 3 years after implementation of retention recommendations had commenced, comparing the findings with the UK experience. It quantifies the levels and explore the characteristics and determinants of trainees’ and non-training scheme doctors’ intentions to emigrate, either temporarily or permanently; and it explores how their migration intentions differ by nationality, age, familial status, training status and stage, and specialty intentions. The paper discusses reasons why implementation of Ireland’s retention strategy has fallen short; and proposes that national medical workforce bodies adopt and implement a suite of differentiated measures, tailored to the intentions, stage and characteristics of their workforces.

## Methods

### Study Design and Sample

The authors designed a quantitative tool to be delivered through an online survey of all NCHDs working in public sector posts in Ireland, utilizing a database maintained by the National Doctor Training and Planning (NDTP) Unit of Ireland’s National Health Service Executive. All doctors taking up a public sector NCHD post are required to enter their details on the database, including demographics, training status and specialty. NCHDs who move to new posts, which occurs typically 6- or 12-monthly, are required to update their details on the database. In November 2017, there were 5260 NCHDs on the database, excluding 733 interns (doctors undertaking their compulsory pre-registration year training, following graduation from medical school). Interns were excluded from this study because they lacked sufficient experience of training and working conditions needed to answer questions on the status of implementation of the Strategic Review recommendations.

### Development of the Survey Tool

The study objective, reported here, was to investigate and report the associations of professional characteristics, demographic factors, and training and working experiences, with 4 possible career intentions: ‘remain in Ireland,’ ‘go abroad but return,’ ‘go abroad and not return,’ or ‘leave medicine.’ Tool developmentwas informed by 2 earlier studies on doctor emigration,^22,23^ and by discussions at the Department of Health-led Strategic Review implementation monitoring group,^37^ on which 2 of the authors sat. This Group met 20 times, February 2015–September 2019 and issued ten progress reports (see https://www.gov.ie/en/collection/9ef920-strategic-review-of-medical-training-and-career-structure-maccraith-/). In 2017, the monitoring group decided to focus efforts on 4 substantive issues “regularly raised by the trainee delegations as requiring specific and urgent attention.”^40^ These were: (*i*) ensuring protected training time for NCHDs; (*ii*) non-core task reallocation, which meant re-allocating basic tasks from NCHDs to other staff – specifically taking blood samples, erecting intravenous lines, discharging patients and giving first doses of medication; (*iii*) covering the costs of training courses and examinations; and (*iv*) controlling the rising numbers and poor conditions of service of doctors in non-training scheme posts, who constitute half of all NCHDs in Ireland.^20,24^

The views of NCHDs on progress on issues (*i*) to (*iii*) were elicited through inclusion of Likert scale questions in the structured questionnaire. Issue (*iv*) was investigated through surveying this growing and hitherto neglected cohort of doctors.^20^ Five other Likert scale questions covered important dimensions of NCHD training and working conditions that were impacting on doctor retention, as reported from published research^22,23,25,26,41^; and a review of 6-monthly consultations with national representatives of trainees, overseen by the Strategic Review monitoring group,^40^ in some of which 2 of the authors participated. Bullying in the workplace was included, because high rates were reported in published studies.^23,41-43^ Level of supervision was included as it predicted trainee intention to emigrate.^23^ Mentoring was included as it formed one of the Strategic Review recommendations; was reported in trainee consultations as ‘generally absent’^40^; and because trainees “… juxtaposed dismissive treatment from certain consultants with the relief they felt with more supportive trainers.”^41^ Two general measures of working conditions were included: ‘staff levels in my workplace,’ which had emerged as a critical factor impacting negatively on training as well as working conditions in a late 2017 national policy dialogue^33^; and ‘level of stress in my working environment.’^44^

The question asked for each item was: “to what extent have the following training/working conditions become better or worse since you began working as a NCHD in Ireland.” Response options were on a 6-point Likert scale ranging from: much better, better, about the same, somewhat worse, much worse and ‘I haven’t been in-place long enough to judge.’ Demographic and training characteristics were included which were known from earlier work to capture the profile of the sample^23^; and were possible predictors for the outcome variables – see Tables 1 and 2. A draft questionnaire was reviewed by training body and NCHD representatives of the Forum of Postgraduate Medical Training Bodies and feedback was incorporated. Piloting was undertaken with 8 NCHDs, 3 of whom were experienced researchers, to review content/acceptability and to trial delivery of the survey through smart phones to encourage completion.

**Table 1:** Frequency and Percentages of Demographic, Training and Career Intention Characteristics

**Variable**	**Value**	**No. (% of Sample)** ^a^	**No. (% of Responders)Excluding Unknowns**
Total		1148 (100%)	
Age(y)^b^	Mean	31	
	Median	30	
	IQR	28–34	
	Range	23–56	
	Unknown	134	
Gender^b^	Female	586 (51%)	586 (56%)
	Male	469 (41%)	469 (44%)
	Unknown	93 (8%)	
Marital status^b^	Married/co-habiting	605 (53%)	605 (57%)
	Single	453 (40%)	453 (43%)
	Unknown	90 (7%)	
Dependents^b^	Yes	251 (22%)	251 (24%)
	No	810 (71%)	810 (76%)
	Unknown	87 (7%)	
Nationality^b^	Irish	751 (65%)	751 (74%)
	Other EU	62 ( 5%)	62 ( 6%)
	Non-EU	206 (18%)	206 (20%
	Unknown	129 (12%)	
Under-grad pathway^c^	DEM	579 (50%)	579 (59%)
	GEM	206 (18%)	206 (21%)
	Not applicable^c^	197 (17%)	197 (20%)
	Unknown^b^	166 (15%)	
Training status	Trainee	752 (66%)	752 (66%)
	Non-trainee	396 (34%)	396 (34%)
Training grade	BST	306 (41%)	306 (41%)
	HST	291 (39%)	291 (39%)
	Run through training	155 (21%)	155 (21%)
	Total trainees	752 (100%)	752 (100%)
Specialty	General practice	84 (7%)	7%
	Surgery	275 (24%)	24%
	Medicine	552 (48%)	48%
	Anesthesiology	122 (11%)	11%
	Psychiatry	110 (10%)	10%
	Unknown	5 ( 0%)	
Career intention	Remain in Ireland	520 (45%)	520 (45%)
	Go but return	399 (35%)	399 (35%)
	Go and not return	196 (17%)	196 (17%)
	Quit Medicine	33 ( 3%)	33 ( 3%)
Migration timing (if leaving)	Before specialist training	122 (21%)	122 (21%)
	During specialist training	122 (21%)	122 (21%)
	After specialist training	338 (58%)	338 (58%)
	Total	582 (100%)	582 (100%)
Intended country of migration (if leaving)	UK	154 (27%)	154 (27%)
	Australia	131 (23%)	131 (23%)
	Canada	125 (22%)	125 (22%)
	USA	54 ( 9%)	54 ( 9%)
	New Zealand	44 ( 8%)	44 ( 8%)
	Other country	64 (11%)	64 (11%)
	Total	572 (100%)	572 (100%)

Abbreviations: DEM, direct entry medicine; BST, Basic Specialist Training; HST, Higher Specialist Training; GEM, graduate entry medicine; EU, European Union; IQR, interquartile range. ^a^Totals = 1148, or less where there were missing data. ^b^Missing data for demographic questions (7%-12%), which were asked at the end of the questionnaire. They represent respondents who did not complete the questionnaire. ^c^The question on undergraduate pathway, DEM or GEM was asked only of respondents who graduated from an Irish medical school, because these models would not be familiar to respondents who had graduated outside of Ireland.

**Table 2:** Associations of Respondent Characteristics With Career Intentions

**Variable**	**Value**	**Remain, No. (%)**	**Go Abroad and Return, No. (%)**	**Go and Stay Abroad, No. (%)**	**Quit Medicine,** **No. (%)**	**Total,** **No.** ^a^ ** (100)**	***P*** **Value** ^b^
Total		520 (45)	399 (35)	196 (17)	33 (3)	1148	<.001
Age (y)	<30	150 (37)	186 (46)	51 (13)	16 (4)	403	<.001^c^
	≥30 <35	167 (42)	140 (35)	78 (20)	14 (4)	399	
	≥35 <40	73 (54)	29 (22)	29 (22)	3 (2)	134	
	≥40	56 (72)	10 (13)	12 (15)	0 (0)	78	
Gender	Female	280 (48)	218 (37)	67 (11)	21 (4)	586	<.001
	Male	192 (41)	154 (33)	111 (24)	12 (3)	469	
Marital status	Married/cohab	295 (49)	176 (29)	110 (18)	24 (4)	605	<.001
	Single	182 (40)	193 (43)	69 (15)	9 (2)	453	
Dependents	Yes	142 (57)	49 (20)	51 (20)	9 (4)	251	<.001
	No	331 (41)	326 (40)	129 (16)	24 (3)	810	
Nationality	Irish	328 (44)	321 (43)	78 (10)	24 (3)	751	<.001^c^
	Non-EU	91 (44)	31 (15)	78 (38)	6 (3)	206	
	Other EU	39 (63)	9 (15)	12 (19)	2 (3)	62	
Undergraduate pathway	DEM	230 (40)	270 (47)	60 (10)	19 (3)	579	<.001
	GEM	102 (50)	64 (31)	36 (17)	4 (2)	206	
	Not applicable	97 (49)	38 (19)	59 (30)	3 (2)	197	
Training status	Non-trainee	205 (52)	90 (23)	89 (22)	12 (3)	396	<.001
	Trainee	315 (42)	309 (41)	107 (14)	21 (3)	752	
Training grade	BST	134 (44)	112 (37)	48 (16)	12 (4)	306	.13^c^
	HST	120 (41)	130 (45)	38 (13)	3 (1)	291	
	Run through	61 (39)	67 (43)	21 (14)	6 (4)	155	
Specialty	General practitioner	53 (63)	20 (24)	8 (10)	3 (4)	84	<.001^c^
	Surgery	124 (45)	89 (32)	51 (19)	11 (4)	275	
	Medicine	240 (43)	207 (38)	89 (16)	16 (3)	552	
	Anesthesiology	41 (34)	60 (49)	19 (16)	2 (2)	122	
	Psychiatry	58 (53)	22 (20)	29 (26)	1 (1)	110	

Abbreviations: DEM, direct entry medicine; GEM, graduate entry medicine; BST, Basic Specialist Training; HST, Higher Specialist Training; EU, European Union. ^a^ Totals for each row = 100% - see Total No. (100%) column. ^b^ The *P* values here represent the probability of the observed data given the assumption of independence of career intentions and demographic variables. For example, H0: Career intention is independent of respondent age, is rejected at the 5% significance level. This particular test has a *P* value <.001. Note: the exclusion of the intention to quit category does not result in any substantial changes to the calculated *P* values. ^c^Simulated Fisher value as chi-squared approximation is not appropriate.

### Data Collection

The NDTP emailed an invitation to all NCHDs in November 2017, on behalf of the MedTrack research team with a link to the questionnaire, hosted on Survey Monkey; a down-loadable information sheet, explaining the nature and purpose of the study; and the researchers’ contact details. National trainee representatives contacted NCHDs to encourage completion. Following 2 reminders, the survey was closed in February 2018, and the NDTP transferred an anonymized dataset to the RCSI researchers.

### Analysis

Analysis was undertaken using the open source statistical software package R: https://www.R-project.org/. The specified outcome (dependent variable) of interest was career intention, with 4 career options (see Methods). Bivariate and multivariable analyses were carried out on these dependent variables using the demographic, training and Likert responses, which were collapsed into 4 categories: better, worse, the same, or new to post. Independent variables with large numbers of values were re-categorized as shown in Table 1, with 11 options for specialty reduced to 5, as categorized by the NDTP. For the migration outcome, a two-stage multiple logistic regression analysis was undertaken. First, associations of predictor variables were compared between respondents who selected the outcome ‘remain in Ireland’ with those who selected an intention to leave Ireland, combining the subcategories ‘go abroad but return’ and ‘go abroad and not return’ ( see Table S3 in Supplementary file 1). Secondly, associations were compared between the 2 ‘leave Ireland’ categories: ‘go return’ versus ‘go not return’ (see Table S4 in Supplementary file 1). Variable reduction in model selection for parsimony was achieved by using a stepwise regression algorithm with bidirectional elimination to identify the most prominent predictive variables for each intention outcome. The choice between competing models was made via Akaike’s information criterion^45^ which penalizes model complexity in favor of simpler explanatory models. For the best fitting models analysis of multi-collinearity via variance inflation factor, analysis showed no grounds for concern (max variance inflation factor = 2.6). All tests of significance are reported at the 95% two-tailed probability level.

## Results

The results are based on the 1148 NCHDs who completed the question: ‘what is your long-term plan in relation to your decision to practice medicine in Ireland?’ – see Tables 1 and 2. The response rate was 22%, which was considered reasonable, given difficulties in achieving high response rates among doctors. A further 320 responses (total 1468) lacked data on migration intentions. The mean age of responders was 31 years; 56% were female; 57% were married or co-habiting; and 24% reported having dependent children (Table 1). Three quarters (74%) were Irish while 20% were non-EU nationals. Among the 785 Irish medical school graduates, who were asked this question, 59% had trained via direct entry medicine (DEM) and 21% via GEM. Two-thirds (66%) of responders were trainees, with similar proportions at earlier and later stages of specialist training. Medicine, at 46%, was the most popular intended specialty; a quarter planned a career in surgery and 8% in general practice. When compared with the NDTP database,^20,21^ this sample contained more trainees (66% versus 51%); females (56% versus 51%), and graduates of Irish medical schools (68% versus 52% – data not tabulated), over-representing these subgroups in the findings.

Close to half of the sample (520, 45%) planned to remain in Ireland; 399 (35%) planned to go abroad but return to Ireland; 196 (17%) planned to go abroad and not return; and 33 (3%) planned to leave medicine. Of those planning to leave Ireland, most(58%) intended to leave after completing specialist training, with 91% selecting 1 of 5 Anglophone destination countries (Table 1).

Table 2 presents the associations of career outcomes with the demographic and training characteristics of the 1148 respondents. Differences in career outcomes were highly statistically significant (*P* < .01) for all but one association. From a workforce perspective, the migration plans of trainees are of particular importance. Trainees were somewhat less likely than non-trainees to remain (42% vs. 52%), but if they were to leave, they were almost twice as likely to return (41% vs. 23%). The proportion of those in HST who intended to ‘go and stay abroad,’ at 13%, was marginally lower than for other trainee grades.

Females were more likely than males to remain in Ireland (48% vs. 41%) and half as likely to stay abroad (11% vs. 24%). Those over 40 years were twice as likely to remain in Ireland (72% vs. 37%) and less than one third as likely (13% vs. 46%) to go abroad and return, compared with those under 30 years. Single NCHDs were more likely than those married/co-habiting to ‘go and return’ (43% vs. 29%), as were those without children. However, if NCHDs who were married or with children left, they were less likely to return. Those planning careers in General Practice were most likely to remain in Ireland (63%); those opting for Anesthesiology were most likely to go abroad with a view to returning to Ireland (49%); whereas those opting for Psychiatry were more likely than other specialties to go and stay abroad (26%). Irish nationals were almost 3 times more likely than non-EU and other-EU nationals to go abroad but return (43% vs. 15%); while other EU nationals were more likely to remain in Ireland (63% vs. 44%). Almost 4 times as many non-EU nationals planned to stay abroad compared to Irish nationals (38% vs. 10%).

Over half (58%) of those planning to leave, including 64% of Irish nationals and 70% of trainees, reported that they would do so after completing specialist training (see Table S1). Non-EU nationals were 3-4 times more likely than others to leave before starting specialist training; and other (non-Irish) EU nationals were 2-3 times more likely to leave during specialist training. Doctors under 30 years were more likely to leave before starting specialist training, with over two-thirds of doctors over 30 years planning to leave after completion. Almost all (93% of) Irish nationals selected 1 of 5 Anglophone countries as a preferred destination country (see Table S2 in Supplementary file 1), with the United Kingdom, Australia and Canada accounting for 72%. Other EU and non-EU nationals especially, were more likely to select the United Kingdom, or other unlisted destination countries; and younger, single and Irish doctors tended to prefer Australia.

Box 1 lists the training and working experience Likert questions posed to respondents. Tables S3 and S4 show the associations of their training and working conditions experiences with their migration choices, firstly comparing doctors planning to remain with doctors planning to leave Ireland (see Table S3 in Supplementary file 1); and secondly, comparing those planning ‘go but return’ with those planning to ‘go and not return’ (see Table S4 in Supplementary file 1). Those who intended to leave rated 7 of 8 dimensions (all except non-core tasks) as significantly worse (*P*≤ .01) compared with those planning to remain. Among those who planned to leave, those planning to ‘go and not return’ rated 6 of 8 dimensions (all except training costs and staffing levels) as significantly worse (*P* < .01), compared with those planning to ‘go but return’ to Ireland. The highest negative ratings among those planning to leave permanently were for ‘stress levels,’ where 62% reported these as worse, followed by ‘staffing levels’ and ‘training costs’ (52%); and ‘protected training’ (48%). Of the 33 doctors who planned to quit medicine, 32 reported their training and work experiences, which were consistently worse than all other respondents: staffing levels and stress levels worse (75%), protected training time (59%), mentoring (56%), training costs (53%), non-core tasks (50%); and 44% reported that bullying had become worse.

**Box 1. Training and Working Conditions**

To what extent have the following training conditions become better or worse since you began training?

- My costs associated with my training
- Protected training time
- Level of supervision of my training
- Mentoring supports within my training programme

To what extent have the following working conditions become better or worse since you began training?

- Non-core task allocation
- Level of bullying in the workplace
- Level of bullying in the workplace
- Staffing levels in my workplace

A two-step multivariable model was constructed, into which the statistically significant independent variables in Table 2, Tables S3 and S4 were entered. The best models are presented in Tables 3 and 4.

**Table 3:** Multivariable Model ORs for “Leave Ireland” Versus “Remain”

**Variable**	**ORs**	**Confidence** **2.5%**	**Intervals** **97.5%**	***P*** ** Value**
Baseline	0.43	0.22	0.81	.01
Age <30 years	1.09	1.05	1.12	<.001
Nationality				
Irish	Reference			
Non-EU	1.54	1.03	2.30	.037
Non-Irish EU	0.63	0.34	1.17	.146
Training status				
Non trainee	*Reference*			
Trainee	1.50	1.06	2.12	.02
Specialty				
General practice	Reference			
Medicine	2.85	1.63	4.98	<.001
Surgery	3.27	1.79	5.98	<.001
Anesthesiology	5.09	2.56	10.10	<.001
Psychiatry	2.60	1.32	5.12	.006
Supervision				
Remains the same	Reference			
New to post	0.82	0.43	1.56	.544
Has become worse	0.87	0.59	1.29	.499
Is better	0.64	0.45	0.91	.014
Training costs				
Remain the same	Reference			
New to post	0.55	0.30	1.00	.051
Has become worse	1.25	0.90	1.73	.183
Are better	0.43	0.23	0.82	.011

Abbreviations: ORs, odds ratios; EU, European Union. Note: The baseline reference individual for odds ratios is a 30 year old non-trainee Irish doctor who intends to specialize in General Practice and who reports training and working experiences (training costs and supervision) as having stayed the ‘same.’

**Table 4:** Multivariable Model ORs for “Stay Abroad” Versus “Return to Ireland”

**Variable**	**OR**	**Confidence** **2.5%**	**Intervals** **97.5%**	***P*** ** Value**
Baseline	0.15	0.04	0.58	.006
Age >30 years	1.16	1.08	1.24	<.001
Nationality				
Irish	Reference			
Non-EU	9.85	4.44	21.86	<.001
Non-Irish EU	3.42	1.03	11.39	.045
Study pathway				
DEM	Reference			
GEM	2.17	1.10	4.28	.026
Entry: not applicable	1.32	0.56	3.09	.526
Specialty				
General practice	Reference			
Medicine	0.67	0.18	2.53	.552
Surgery	0.71	0.18	2.85	.631
Anesthesiology	1.04	0.25	4.27	.954
Psychiatry	4.76	1.05	21.56	.043
Bullying				
Remains the same	Reference			
New to post	0.38	0.11	1.31	.124
Has become worse	0.95	0.49	1.84	.890
Is better	0.46	0.21	0.99	.047
Mentoring				
Remains the same	Reference			
New to post	1.30	0.40	4.25	.660
Has become worse	5.85	2.97	11.54	<.001
Is better	0.77	0.39	1.52	.446

Abbreviations: ORs, odds ratios; DEM, direct entry medicine; GEM, graduate entry medicine; EU, European Union. Note: The baseline reference individual is a 30 years or younger Irish doctor who intends to do general practice, who entered medicine as a direct entry student and who reported prevalence of bullying and quality of mentoring as having stayed the same.

The first model (Table 3) compares ‘leave Ireland’ versus ‘remain in Ireland.’ The characteristics independently statistically significant for those planning to leave Ireland were: age less than 30 years (odds ratio [OR] = 1.09 per year under 30; *P* < .001); non-EU nationality (OR = 1.54; *P* = .037); and being a trainee (OR = 1.50; *P* = .022). General Practice (least likely to leave) was the reference point for specialty intentions, where the highest OR for leaving was among those planning to specialize in Anesthesiology (OR = 5.09; *P* < .001). Those opting for all other hospital specialties were also significantly more likely to leave. Those reporting supervision and training costs as ‘better’ were significantly more likely to remain (*P* < .05).

Table 4 shows the second step multivariable model, comparing ‘go and not return’ versus ‘go but return to Ireland,’ among respondents who planned to leave. Here, older NCHDs (age corrected to a baseline of 30 years or less) were more likely to stay abroad (OR = 1.16 per year of age > 30; *P* < .001), as were non-EU (OR = 9.85; *P* < .001) and non-Irish EU (OR = 3.42; *P* = .045) nationals, GEM graduates (OR = 2.17; *P* = .03), and those specializing in Psychiatry (OR = 4.76; *P* = .043). There was a strong, significant association between experiencing mentoring as ‘worse’ and an intention to remain abroad (OR = 5.86; *P* < .001); and those who reported bullying as ‘better’ were significantly more likely to return (OR = 0.47; *P* = .047).

In a multivariable model for ‘leave medicine’ versus ‘continue in medicine’ (see Table S5 in Supplementary file 1), the 32 doctors who responded to the Likert questions and who planned to quit medicine were significantly more likely to be younger (OR = 0.88 per year over 30 years old; *P* = .014); married or co-habiting (OR = 2.98; *P* = .01); and significantly more likely to report mentoring as worse (OR = 4.55; *P* = .001).

## Discussion

From the perspective of a national sample of 1148 NCHDs, national retention measures^37,39^ have failed to effectively address stressful working conditions and unsatisfactory training. This has meant, from a national medical workforce policy perspective, that the benefits of increased domestic production of doctors have not been realized^18^; and Ireland’s compliance with the cornerstone principle of the WHO Global Code – ‘train and retain’ – has been undermined through largescale doctor emigration.^25,26^ The 2018 report, *Recruitment and Retention of the Health Workforce in Europe*, proposed 5 categories of interventions: education, regulation, financial incentives, professional and personal support, and a mix of such interventions.^46^ The 25 recommendations to improve doctor retention, agreed 4 years previously as the blueprint for doctor retention in Ireland,^37^ had resulted in some fairly easy ‘wins’ in the areas of professional and personal supports for doctors, and education and training for NCHDs.^39^ Successive monitoring reports show better communication and career planning supports for NCHDs; reduced paper work when they move post; more predictability around training locations; and access to advanced fellowship training in Ireland, enabling some doctors to complete specialist training at home.^39^ However, the root causes of the unsatisfactory training and working conditions that are driving NCHD emigration have not been adequately addressed.

Three of the 4 *training* dimensions – protected training, supervision, mentoring supports, which were more likely to be reported as getting worse by doctors who planned to leave (worse still if leaving permanently), are directly dependent on there being sufficient consultant trainers. Implementation of all four 2014 Strategic Review recommendations, aiming to make consultant posts more attractive, had been deemed successful.^39^ However, the failure of Government to address the two-tier consultant contract, introduced in 2012 as a response to economic austerity, has contributed to loss of salary competitiveness vis-a-vi other high-income Anglophone countries, resulting in an estimated 500 consultant posts being unfilled or occupied by locums.^47-49^ This is a major deterrent to consultant recruitment, especially of Irish trainees exiting specialist training.^48^ Unfortunately, we did not capture NCHDs’ views on the two-tier consultant contract in this study. Two of the 4 *working conditions* factors associated with an intention to leave – worse staffing levels and excessive non-core tasks – point to a broader failure of health workforce allocation to meet increasing demands for healthcare.

Doctors’ experiences of worsening training and working conditions are not unique to Ireland. Findings from a 2018 General Medical Council report show that around one quarter to a half of UK doctors report working longer hours, a worsening work-life balance, a lack of support from senior colleagues, and worsening mentoring experiences.^11^ The UK report shows lower annual exit rates among younger UK medical graduates, at 2.6% under 30 years, compared with 6.0% among 25-34 year Irish graduates.^24^ It shows a similar reliance on, but proportionately higher exit rates among, international medical graduates (UK = 5.7%, Ireland = 8.6-11.4%).^11,24^ Unlike our paper, however, the UK report does not link migration plans with specific experiences. The findings in this paper will not surprise medical workforce stakeholders in Ireland; but do shed light on and help to quantify some of the drivers of doctor emigration from an Anglophone high-income country. From a medical workforce strategy perspective, a more differentiated understanding of the characteristics and preferences of doctors who plan to remain, to leave and return, or to leave permanently, to which this paper contributes, can inform the design of a more tailored suite of retention measures.

The typical doctor who intends to leave Ireland but return later is under 30 years, male, single, has no dependents, is Irish and is training for a hospital specialty, similar to the ‘back packer’ category in Glinos and Buchan’s framework.^50^ Australia is his preferred destination country, which is consistent with published studies.^25,26^ Globalization and the highly portable nature of medical qualifications, especially from an Anglophone country, mean that such outward migration by early-career doctors will continue. The challenge for Ireland lies in getting these doctors back to make their careers in Ireland.^28,32^ A positive finding, in respect to retention measures, was that a perception that supervision and training costs had improved were associated with an intention to remain. A negative finding was that trainees were more likely than non-trainees to leave (55% versus 45%); and most (70%) planned to leave after they completed their specialist training, at a point when they are highly sought after for permanent posts by employers abroad, and when most of the costs of their training has been borne by Irish taxpayers. Ireland has a long tradition of its trainees undergoing specialty and super-specialty higher level training in centers of excellence,^25,26^ especially in the United Kingdom, the United States and Canada, which were the preferred destination countries for most NCHDs in HST. However, as questioned by Humphries and colleagues, training in centers of excellence abroad may not always be appropriate to the needs of the Irish health system^25,26^; and may lead to permanent emigration.^22,28,32^

The typical doctor who intends *not* to return to Ireland is over 30 years, male and non-Irish; is married/co-habiting, has dependent children; and is a GEM graduate. The strong association with a negative experience of mentoring suggests that some dimension of personal relationship or support from individual trainers or senior colleagues is an important determinant of an early career doctor’s intention to make a long-term career in the country where s/he trained, consistent with earlier qualitative findings.^41^ Specialty-specific associations – those planning careers in Anesthesiology are more likely to leave and return whereas permanent emigration is more likely in Psychiatry – require further exploration. Non-EU nationals are more likely to leave and almost ten times more likely to not return, which an earlier study reported as being due to dissatisfaction with working conditions, lack of training and career opportunities in Ireland.^51,52^ Hence, this body of research confirms that international recruitment is only a stopgap solution; and is an ineffective strategy for staffing a country’s health services. Non-EU nationals were looking to the United Kingdom, where they were likely to have better opportunities of getting into training programs.^11^ Whereas, almost two-thirds of non-Irish EU nationals, many of whom had graduated from central European country medical schools,^24^ envisaged remaining in Ireland.

The percentage of respondents planning to quit medicine, at 3%, was small, compared with 7% of UK graduates, aged 35-54 years, who reported that this is ‘the main career change they are considering.’^11^ However, the 32 doctors who responded to these questions reported worse experiences for all training and working factors than did all other respondent categories who were continuing in medicine. While an etiological link cannot be assumed from a cross-sectional study, the results suggest that poor training and working conditions are driving early career doctors to leave Ireland, often for good; and in a small number of cases to quit medicine. An intention to quit medicine has previously been associated with self-reported burnout and the need for better support from senior colleagues.^53^ Lower rates quitting medicine but higher rates leaving Ireland, compared with the United Kingdom, suggest that emigration provides an escape-hatch from intolerable working conditions.^22,28^ Further publications from Humphries and colleagues may throw light on the comparative advantages of working and training conditions experienced by Irish-trained doctors abroad.^25^

## Policy Implications

The Strategic Review of Medical Training and Career Structures implementation monitoring group – known as the MacCraith Group^37^ – met 22 times by March 2020 and produced 10 progress reports since its inception in January 2015. After 5 years implementation, reasons for successes – but also the failure of the national response to impact on under-staffing, stressful working conditions and the displacement of training by service demands on NCHDs, as reported in this paper – are apparent. Firstly, there is the monitoring group’s composition, which has been identified as a key feature of successful implementation of retention measures.^46^ Strengths of the monitoring group include Department of Health leadership, and membership that includes national training bodies, trainee and medical professional representatives; and also 2 key units of Ireland’s National Health Service Executive – NDTP and Human Resources.^38^ As a result, implementation of education and training measures, and professional and personal supports, as described earlier, could be achieved. However, the multi-faceted set of 25 interventions^37^ to improve retention was a mixed blessing: it meant that many smaller problems were tackled, but there was a lack of focus and impact on the substantive issues driving emigration.

The study findings in this paper, which demonstrate different factors and characteristics distinguishing doctors who are considering temporary versus permanent emigration, point to the need for a diversified approach to doctor retention. Younger trainees, especially if aiming for a hospital specialty, are more likely to train in Ireland if supervision needs and training cost obstacles are addressed. Given that a period of living and training abroad may bring personal and professional awards,^25,26^ Irish employers should also focus on making timely offers and ensuring efficient appointment systems for those who are eligible to take up hospital consultant posts in Ireland, as they complete their specialty training abroad.^23^ However, while better management of career pipelines may promote more efficient circular migration, the types of doctors and factors leading to a minority (17%) of doctors planning to leave and not return call for a different set of responses. Study findings point to the potential benefit of early establishment of strong mentoring links between senior specialists and trainees, and maintenance of those mentoring links during periods of training abroad, to encourage trainees to make their careers in Ireland.

The findings confirm earlier studies that pointed to the likelihood of non-Irish doctors seeing Ireland as a stepping stone to careers elsewhere^51,52^; and the need for Ireland to dispense with international recruitment as a medical workforce strategy. Irish medical workforce policy has been undermined by the European Working Time Directive,^54^ whose intent, in the health sector, was to limit the number of hours on-call and improve the lives of hospital doctors. Its unintended consequence was to drive recruitment of the only type of doctors willing to work in Ireland’s smaller hospitals, which require 24-hour cover, even if relatively few patients access their acute care services – international medical graduates. This has reversed efforts to reduce and eliminate non training scheme posts, whose numbers increased from 900 in 2013 to 2724 in 2018 – mainly international graduates, virtually equaling the number of training posts (2779).^20^ A multi-stakeholder dialogue, hosted by 2 of the authors, concluded that NCHD posts in such smaller hospitals are difficult to fill because they are ineligible and unsuitable for training, detracting from efforts to fill consultant posts in these hospitals.^33^ Some of the retention measures that are needed – to address hospital under-staffing and consultant shortages – required the allocation of resources, over which the national Strategy Implementation Monitoring Group had little influence.

Ireland is a microcosm of global developments, where the demand for and on doctors is growing inexorably. There will be an estimated shortfall of 750 000 doctors in 31 OECD countries by 2030, excluding an estimated shortage of 2.6 million doctors in poorer countries, from which countries like Ireland recruit their doctors.^55^ Demographic change (older populations), increasing complexity of care and rising expectations and demands from patients are putting huge pressures on doctors, in all countries, contributing to overwork, burnout and poor mental health, linked to large numbers of doctors exiting the profession.^11^ Ireland can at least claim that it is getting a handle on the size of the problem. Burnout and psychological ill health have been reported by around one third of hospital specialists and trainees in national surveys^55,57^; and self-reported burnout, callousness and negative experiences among recent graduates were associated with an intention to go abroad and not to return to Ireland.^30^ Also, high levels of bullying – experienced by a third and observed by a half of trainees in recent surveys^41,43^ – may partly be a consequence of stress. Initiatives by training bodies and employers to improve training experiences and working conditions will have limited effect in the absence of sufficient political will and resources to ensure an effective response to Ireland’s medical workforce crisis. A policy framework exists to tackle this chronic, national medical workforce crisis – the 2017 *Sláintecare* Report – which proposes radical increases in general practitioner and hospital specialist numbers,^58,59^ given that some specialties are currently one third the ratio-to-population found in other countries.^33^ Young Irish doctors started to return home in their hundreds in March 2020 to support the response to the COVID-19 epidemic.^60^ The question now is, once the epidemic is under control: will Ireland take the steps to keep them?

## Acknowledgements

The authors acknowledge and thank the doctors who participated in the survey and the NDTP unit of Ireland’s national Health Service Executive, which facilitated survey administration.

## Ethical issues

This study was reviewed and approved by the Royal College of Surgeons Research Ethics Committee (REC 1435).

## Competing interests

RB is a member of the Strategic Review of Medical Training and Career Structures Implementation Monitoring Group. Nothing in this paper can be attributed in any way to the Monitoring Group or any participating agency or member of the Group.

## Funding

This study has been funded by a Health Research Board Grant (grant number HRA-HSR-2015-1304).

## Authors’ contributions

RB had lead responsibility for study conception and design, data interpretation, obtaining funding and led on drafting of the manuscript. NC had joint responsibility for study conception and design, was co-lead of data acquisition, and undertook critical revision of the manuscript. LH contributed to study conception and design, was co-lead of data acquisition, and undertook critical revision of the manuscript. JS had lead responsibility for statistical analysis, contributed to data interpretation, and undertook critical revision of the manuscript.

## Authors’ affiliations

^1^RCSI Division of Population Health Sciences, Royal College of Surgeons in Ireland, Dublin, Ireland. ^2^School of Psychology, Dublin City University, Dublin, Ireland. ^3^National Doctors Training and Planning, Health Service Executive, Dublin 8, Ireland. ^4^Department of Mathematics & Statistics, University of Limerick, Limerick, Ireland.

## Supplementary files

Supplementary file 1. contains Tables S1-S5.

## Key Messages

**Implications for policy makers**

- Ireland is failing to retain its own medical graduates, many of whom – especially trainees – intend to leave to work in other Anglophone countries, undermining Ireland’s adherence to a principle of the World Health Organization (WHO) Global Code on the International Recruitment of Health Personnel.
- Implementation of Ireland’s 2014 national doctor retention strategy achieved some successes in education and training but did not tackle the root causes of outward migration, related to low staffing levels, poor training experiences and stressful working conditions.
- Poor supervision experiences and training costs distinguish leavers and stayers. Bullying in the workplace, and above all a strong association with worsening mentoring experiences, distinguish doctors who will remain abroad from returners.
- A more diversified retention strategy is needed, facilitating career paths towards toward permanent posts in Ireland for younger trainees who choose to undertake specialty training abroad.
- Specialty-specific migration-drivers need to be identified and addressed; measures to combat bullying reinforced; and mentoring links between trainees and senior specialists need to be strengthened to counteract the negative experiences of training in Ireland.

**Implications for public**

Ireland’s failure to retain the doctors that it trains means that it has to recruit large numbers of doctors internationally, usually from poorer countries. This paper reports the reasons for this largescale outward migration of doctors, which is largely because of a failure to address poor training experiences and working conditions. The loss to Ireland of this high value resource is the result of not effectively addressing the reasons why doctors leave. Most Irish medical graduates wish to make their careers in Ireland; but many will leave and not return unless better training, working conditions and career opportunities are available. Government needs to invest in the medical workforce so that our medical graduates can train and have careers in Ireland, enabling them to provide high quality care to patients.
