# Supplementary material for: Doctor Retention: A Cross-sectional Study of How Ireland Has Been Losing the Battle
Source: Int J Health Policy Manag. 2020 Apr 22;10(6):299–309. doi: 10.34172/ijhpm.2020.54 (PMC9056149; doi:10.34172/ijhpm.2020.54)
Supplement: Supplementary file 1 — contains Tables S1-S5. [file ijhpm-10-299-s001.pdf]

Supplementary file 1

Table S1. Timing of non-consultant hospital doctors' migration plans

Timing<br>**Variable**	**Go before specialist training ** No. (%)**	**Go during specialist training No. (%)**	**Go after specialist training No. (%)**	**TOTAL No. (100%)**	**P value**
**TOTAL**	122 (21%)	122 (21%)	338 (58%)	582	<0.001
**Nationality**					
Irish	54 (14%)	88 (22%)	256 (64%)	398	
Other EU	2 (10%)	10 (48%)	9 (43%)	21	
Non-EU	47 (45%)	14 (13%)	44 (42%)	105	<0.001*
**Age:**					
< 30 years	64 (27%)	61 (26%)	111 (47%)	236	
≥ 30 < 35	33 (15%)	38 (18%)	145 (67%)	216	
≥ 35 < 40	5 (9%)	11 (20%)	39 (71%)	55	
≥ 40	4 (19%)	2 (10%)	15 (71%)	21	<0.001*
**Gender**					
Male	55 (19%)	62 (22%)	167 (59%)	284	
Female	57 (22%)	49 (19%)	153 (59%)	259	0.6
**Marital status**					
Married/co-habiting	49 (17%)	55 (20%)	177 (63%)	281	
Single	61 (23%)	59 (23%)	140 (54%)	260	0.08
**Entry route**					
DEM	51 (16%)	74 (23%)	203 (62%)	328	
GEM	16 (16%)	16 (16%)	68 (68%)	100	
Not applicable	27 (29%)	18 (19%)	48 (52%)	93	0.02
**Training status**					
Trainee	36 (9%)	88 (21%)	286 (70%)	410	
Non-trainee	86 (50%)	34 (20%)	52 (30%)	172	<0.001
**Training level**					
BST	34 (22%)	49 (31%)	75 (47%)	158	
HST	0 (0%)	33 (20%)	133 (80%)	166	
Run through	2 (2%)	6 (7%)	78 (91%)	86	<0.001
**Specialty**					
GP	1 (4%)	1 (4%)	26 (93%)	28	
Surgery	27 (20%)	22 (16%)	87 (64%)	136	
Medicine	84 (29%)	77 (27%)	129 (44%)	290	
Anaesthesia	3 (4%)	8 (10%)	66 (86%)	77	
Psychiatry	7 (14%)	14 (28%)	29 (58%)	50	<0.001

Abbreviations: DEM, direct entry medicine; GEM, graduate entry medicine; BST, Basic Specialist Training; HST, Higher Specialist Training; EU, European Union; GP, general practitioner.

* Fisher test used as chi square approximation is not appropriate.

For the reported p values the null hypothesis is *H0: the timing of emigration is independent of the variable categorisations/levels for each variable of interest.*

Table S2. Associations of demographic and training characteristics with preferred destination countries

Destination Country<br>Variable	UK<br>No. (%)	Australia<br>No. (%)	Canada<br>No. (%)	USA<br>No. (%)	NZ<br>No. (%)	Country Other<br>No. (%)	TOTAL	P value *
**TOTAL**	154 (27%)	131 (23%)	125 (22%)	54 (9%)	44 (8%)	64 (11%)	572	<0.001
**Nationality**								
Irish	87 (22%)	102 (26%)	95 (24%)	44 (11%)	35 (9%)	26 (7%)	389	
Other EU	7 (33%)	2 (10%)	6 (29%)	0 (0%)	1 (5%)	5 (24%)	21	
Non EU	44 (42%)	10 (10%)	10 (10%)	6 (6%)	6 (6%)	28 (27%)	124	<0.001**
**Age:**								
< 30 years	53 (23%)	68 (30%)	50 (22%)	22 (10%)	21 (9%)	14 (6%)	228	
≥ 30 < 35	65 (30%)	41 (19%)	53 (25%)	18 (8%)	9 (4%)	29 (13%)	215	
≥ 35 < 40	15 (26%)	7 (12%)	7 (12%)	8 (14%)	10 (18%)	10 (18%)	57	
≥ 40	6 (27%)	5 (23%)	2 (9%)	1 (5%)	1 (5%)	7 (32%)	22	<0.001**
**Gender**								
Female	76 (27%)	64 (23%)	74 (27%)	24 (9%)	21 (8%)	18 (6%)	277	
Male	68 (26%)	56 (22%)	43 (17%)	26 (10%)	22 (9%)	42 (16%)	257	0.003
**Marital status**								
Married/co-hab	83 (30%)	50 (18%)	63 (23%)	21 (8%)	21 (8%)	40 (14%)	278	
Single	57 (23%)	72 (28%)	53 (21%)	28 (11%)	22 (9%)	21 (8%)	253	0.009
**Entry route**								
DEM	75 (23%)	85 (27%)	82 (26%)	33 (10%)	23 (7%)	22 (7%)	320	
GEM	16 (16%)	22 (22%)	26 (26%)	13 (13%)	14 (14%)	8 (8%)	99	
Not applicable	39 (42%)	10 (11%)	11 (12%)	4 (4%)	6 (6%)	23 (25%)	93	<0.001
**Training status**								
Non -Trainee	72 (42%)	30 (18%)	12 (7%)	12 (7%)	17 (10%)	28 (16%)	171	
Trainee	82 (20%)	101 (25%)	113 (28%)	42 (10%)	27 (7%)	36 (9%)	401	<0.001
**Training level**								
BST	24 (16%)	46 (30%)	32 (21%)	19 (12%)	17 (11%)	15 (10%)	153	
HST	46 (28%)	27 (16%)	56 (34%)	18 (11%)	4 (2%)	13 (8%)	164	
Run through	12 (14%)	28 (33%)	25 (30%)	5 (6%)	6 (7%)	8 (10%)	84	<0.001
**Specialty**								
GP	1 (4%)	7 (26%)	16 (59%)	0 (0%)	2 (7%)	1 (4%)	27	
Surgery	39 (28%)	33 (24%)	16 (12%)	16 (12%)	12 (9%)	21 (15%)	137	
Medicine	80 (28%)	59 (21%)	70 (25%)	29 (10%)	20 (7%)	24 (9%)	282	
Anaesthesia	26 (34%)	24 (31%)	7 (9%)	4 (5%)	7 (9%)	9 (12%)	77	
Psychiatry	8 (17%)	8 (17%)	16 (33%)	4 (8%)	3 (6%)	9 (19%)	48	<0.001**

Abbreviations: DEM, direct entry medicine; GEM, graduate entry medicine; BST, Basic Specialist Training; HST, Higher Specialist Training; EU, European Union; GP, general practitioner.

* The P values here represent the probability of the observed data given the assumption of independence of career intentions and demographic variables.

** Fisher exact test used as chi-square approximation is not appropriate.

Table S3. Associations of training and working experiences with migration plans: Remain versus Leave

Training and working experiences	Better No. (%)	Worse No. (%)	New to post No. (%)	Same No. (%)	TOTAL No. (100%)	P value
**1. Training costs**						
Remain Ireland	49 (10)	200 (39)	92 (18)	167 (33)	508	
Leave Ireland	28 (5)	272 (47)	73 (13)	203 (35)	576	<0.001
**2. Protected training**						
Remain Ireland	118 (23)	145(28)	77(15)	169(33)	509	
Leave Ireland	97 (17)	206(36)	57(10)	216(38)	576	<0.001
**3. Supervision**						
Remain Ireland	161 (32)	90 (18)	73 (14)	184 (36)	508	
Leave Ireland	130 (23)	118 (20)	61 (11)	268 (46)	577	<0.001
**4. Mentoring supports**						
Remain Ireland	168 (33)	86 (17)	84 (17)	171 (34)	509	
Leave Ireland	152 (26)	126 (22)	65 (11)	234 (41)	577	<0.001
**5. Non-core tasks**						
Remain Ireland	107 (21)	137 (27)	54 (11)	213 (42)	511	
Leave Ireland	109 (19)	167 (29)	44 (8)	252 (44)	572	0.28
**6. Stress levels**						
Remain Ireland	100 (20)	205 (40)	42 (8)	161 (32)	508	
Leave Ireland	75 (13)	287 (50)	32 (6)	182 (32)	576	0.001
**7. Bullying**						
Remain Ireland	126 (25)	81 (16)	63 (12)	238 (47)	508	
Leave Ireland	111 (19)	124 (22)	51 (9)	290 (50)	576	0.007
**8. Staffing levels**						
Remain Ireland	93 (18)	208 (41)	41 (8)	167 (33)	509	
Leave Ireland	72 (12)	283 (49)	38 (7)	183 (32)	576	0.01

* totals for each row = 100%

Table S4. Associations of training and working experiences with migration plans: Go but Return Versus Go and Not Return

Migration level 1	Better<br>No. (%)	Worse<br>No. (%)	New to post<br>No. (%)	Same<br>No. (%)	TOTAL<br>No. (100%)	P value
**1. Training costs**						
Go but Return	18 (5)	175 (45)	55 (14)	140 (36)	388	
Go Not Return	10 (5)	97 (52)	18 (10)	63 (34)	188	0.31
**2. Protected training**						
Go but Return	74(19)	116(30)	42(11)	156(40)	407	
Go Not Return	3(12)	90(48)	15(8)	60(32)	191	<0.001
**3. Supervision**						
Go but Return	90 (23)	57 (15)	47 (12)	194 (50)	388	
Go Not Return	40 (21)	61 (32)	14 (7)	74 (39)	189	<0.001
**4. Mentoring supports**						
Go but Return	114 (29)	51 (13)	49 (13)	174 (45)	388	
Go Not Return	38 (20)	75 (40)	16 (8)	60 (32)	189	<0.001
**5. Non-core tasks**						
Go but Return	84 (22)	97 (25)	33 (9)	172 (45)	386	
Go Not Return	25 (13)	70 (38)	11 (6)	80 (43)	186	0.006
**6. Stress levels**						
Go but Return	59 (15)	169 (44)	25 (6)	134 (35)	387	
Go Not Return	16 (8)	118 (62)	7 (4)	48 (25)	189	<0.001
**7. Bullying**						
Go but Return	81 (21)	64 (17)	38 (10)	204 (53)	387	
Go Not Return	30 (16)	60 (32)	13 (7)	86 (46)	189	<0.001
**8. Staffing levels**						
Go but Return	49 (13)	184 (48)	28 (7)	126 (33)	387	
Go Not Return	23 (12)	99 (52)	10 (5)	57 (30)	189	0.66

Table S5. Multivariable model odds ratios for 'leave' (quit) versus 'continue' in medicine'

Variable	Odds ratio	Confidence 2.5%	Intervals 97.5%	P value
Baseline	1.08	0.04	29.5	= 0.965
Age > 30 years	0.88	0.79	0.97	= 0.014
**Relationship Status:** Single				
Married/ Co-habiting	2.98	1.3	8.82	= 0.01
**Mentoring**: Remains the same				
New to post	0.65	0.13	3.16	= 0.596
Has become worse	4.55	1.92	10.74	= 0.001
Is better	0.61	0.18	2.06	= 0.429

Footnote: The baseline reference individual is a 30 years or younger doctor who is single and who reported the quality of mentoring as having stayed the same.
